# Determinants of Exposure to Environmental Tobacco Smoke (ETS) among Non Smoking Adolescents (Aged 11–17 Years Old) in Greece: Results from the 2004–2005 GYTS Study

**DOI:** 10.3390/ijerph7010284

**Published:** 2010-01-21

**Authors:** George Rachiotis, Seter Siziya, Adamson S. Muula, Emmanuel Rudatsikira, Panagiotis Papastergiou, Christos Hadjichristodoulou

**Affiliations:** 1 Department of Hygiene and Epidemiology, Medical Faculty, University of Thessaly, 6 Lapithon str, Larissa, Greece; E-Mails: gsrachmed@yahoo.com (G.R.); panpapast@med.uth.gr (P.P.); 2 Department of Community Medicine, University of Zambia, Medical School, Box 50110, Lusaka, Zambia; E-Mail: ssiziya@yahoo.com; 3 Department of Public Health, Division of Community Health, College of Medicine, University of Malawi, Private Box 360, Blantyre, Malawi; E-Mail: muula@email.unc; 4 Graduate School of Public Health, San Diego State University, CA 92182, USA; E-Mail: erudatsi@mail.sdsu.edu

**Keywords:** Environmental Tobacco Smoke, adolescents, Greece

## Abstract

The aim of the study is to investigate the determinants of exposure to ETS among Greek adolescents aged 11–17 years old. The GYTS questionnaire was completed by 5,179 adolescents. About 3 in 4 responders (76.8%) were exposed to ETS at home, and 38.5% were exposed to ETS outside of the home. Gender, age group, parental and close friends smoking status were significant determinants of adolescent’s exposure to ETS. The results of the study could be valuable for the implementation of public health initiatives in Greece aiming to reduce the burden of adolescent’s exposure to passive smoking.

## 1. Introduction

Tobacco use is a significant preventable cause of disability, and premature death at a worldwide level. Nearly five million persons die annually from tobacco-related illnesses, and many more suffer from smoking-related morbidity. Furthermore, the number of fatalities is expected to more than double by year 2020 [1]. The adverse effects of exposure to Environmental Tobacco Smoke (ETS) on health of adolescents are well known and include increased risk for asthma induction and exacerbation, acute lower respiratory tract infections, and effusions of the middle-ear. Exposure to ETS is associated with abnormal levels of lung function, and increased bronchial responsiveness in both adults, and adolescents [2–4]. Besides the effects of ETS on health, exposure to ETS could be associated with significant economic costs due to increased health care services utilization [5]. The Global Youth Tobacco Survey (GYTS) is a school-based survey. The GYTS project is intended to enhance the capacity of countries to develop and evaluate tobacco control and prevention programs. World Health Organization and Centre for Disease Control and Prevention Atlanta, USA have played a leading role in the development of this project [6,7].

## 2. Methods

Our study involved the secondary analysis of the Global Youth Tobacco Survey (GYTS) conducted in Greece among middle-school students in Greece, 2004−2005. A comprehensive description of the data collection methodology was reported previously by Kyrelsi *et al*. [8]. In brief, a two-stage cluster sampling design was instituted in which in the first phase all schools containing the middle-school grades in Greece were identified and 100 schools were selected (25 schools from each region). This was considered adequate to obtain a sample design that would produce representative estimates for each region. In the first stage of sampling, the probability of schools being selected was proportional to the number of students enrolled in the specified grades (grades 1–3 at all middle schools). In the second sampling stage, classes within the selected schools were randomly selected. All students in selected classes attending school on the day of the survey were eligible to participate. The median age of the studied population was 14 years old.

### Data collection

The GYTS questionnaire included data on demographic variables and experience with cigarette smoking. Self-completed questionnaires were used. A project coordinator supervised the data collection process and reported to the supervisor on a daily basis. Completed questionnaires were sent to the Centers for Disease Control and Prevention for processing where they were transformed into electronic files.

### Statistical analysis

A weighting factor was used in the analysis to reflect the likelihood of sampling each student and to reduce bias by compensating for differing patterns of non response. The weight used for estimation is given by the following formula:

W=W1*W2*f1*f2*f3*f4

where W1 = the inverse of the probability of selecting the school; W2 = the inverse of the probability of selecting the classroom within the school; fl = a school-level non response adjustment factor calculated by school size category (small, medium, large); f2 = a class-level non response adjustment factor calculated for each school; f3 = a student-level non response adjustment factor calculated by class; and f4 = a post stratification adjustment factor calculated by grade.

We conducted logistic regression analysis using SUDAAN software version 9.0 (Research Triangle Institute, Research Triangle Park, NC, USA) to estimate associations between relevant predictor variables and ETS. To assess environmental tobacco smoke exposure at home participants were asked: “How often do you see your father/mother/sister/friend/other people smoking in your home?” Four grades of exposure have been used: Don’t have/don’t see this person; about every day; Sometimes; Never. To assess environmental tobacco smoke exposure outside home participants were asked: “How often do you see people smoke in your presence in places other than in your home?”. Three grades of exposure have been set: About every day/Sometimes/Never.

We report unadjusted Odds Ratios (OR) for selected predictor variables while considering exposure to environmental tobacco smoke as dependent variable. We thereafter report results of adjusted odds ratios (AOR) for the factors.

## 3. Results

Table 1 indicates that 5,179 nonsmokers, of whom 49.2% were females, participated in the study. Overall, 79.3% of responders were exposed to ETS at home, 38.2% were exposed to ETS outside of the home. The majority of the participants (89.0%) were in favor of banning smoking in public places.

Table 2 reports the variables associated with ETS exposure in univariate, and multivariate analysis. Compared to participants aged 11−13 years, those aged 15 years or older were more likely to be exposed to ETS at home (OR = 1.54; 95% CI [1.27, 1.86] for participants aged 15 years and OR = 1.37; 95% CI [1.00, 2.05] for participants aged 16–17 years) and less likely to be exposed to ETS outside of the home (OR = 0.68; 95% CI [0.58, 0.79] for responders aged 15 years and OR = 0.58; 95% CI [0.42, 0.81] for those aged 16−17 years). Males were less likely to be exposed to ETS at home (OR = 0.72; 95% CI: 0.64, 0.82). Responders whose parents and close friends were smokers were more likely to be exposed to ETS at home and outside of the home.

Table 2 indicates that the results from multivariate analysis were unchanged for male participants and those whose parents and close friends were smokers. Compared to responders aged 11−13 years, those who were 15 years old or older were more likely to be exposed to ETS at home and less likely to be exposed to ETS outside of the home.

## 4. Discussion

Our results indicated that Greek student’s exposure to secondhand smoke was high, both at home and in public places (79.3%, and 38.2%, respectively). A cross country comparison (within the GYTS project) reported an exposure to ETS at home which varied from 16% in Malawi to 79.8% in India (median: 48.9%). In addition, exposure to secondhand smoke in public places had a range from 30.4% in Malawi to 86.7% in Argentina (median: 60.9%) [7].This wide variation could be attributed to factors like different tobacco control strategies, different cultural and religious norms, differential availability of tobacco products. The present study indicates that the prevalence of student’s exposure to second hand smoke in public places is 38.2%. At a face value this information does not correspond with that in Fact Sheet of Greek Global Tobacco Survey. In particular, the fact sheet indicates that 94% of the students are exposed to passive smoking at home while the rate provided by our data is 79.3%. This is also the case for passive smoking in public places. Our data indicate a rate notably lower to that of Fact Sheet (38.2% *versus* 94%, respectively). The reason for these differences may be that the fact sheet reports for ages 13−15 years old only. On the contrary we report on everyone participant. However taking into consideration only the data of the present study it is of interest that the reported exposure to passive smoking outside home seems to be considerably lower when compared to exposure at home (38.2% *versus* 79.3%, respectively). It is difficult to interpret this finding. However, we can speculate that either students purportedly avoid exposure to second hand smoke in public places, or they underreported passive smoking outside the home.

In Multivariate analysis age, gender, parents and peers smoking status were significant determinants of exposure to ETS in the current study. In particular, our results indicated an age-related pattern of adolescent’s exposure to ETS: Students at age ≥15 years had an increased risk of exposure to ETS at home in comparison to younger adolescents. This finding is in line with that of Rudatsikira *et al*. among adolescents in Mongolia who reported that increasing age was associated with higher likelihood of being exposed to environmental tobacco smoke in both sexes [9]. However, the finding contradicts that of Preston *et al*. among Puerto Rican children who reported a non significant trend [10]. A possible interpretation for the above finding could be that parents are very reluctant to smoke at home in the presence of their youngest children. It is also of interest that adolescents at age ≥15 years were less likely to be exposed to ETS outside of home. It seems plausible that these students (because of the increased risk of exposure to ETS at home) purposively avoid exposure to passive smoking outside of home.

Regarding gender, the results of our study do show that males were less likely to be exposed to ETS than females at home; and were equally likely as females to be exposed to ETS outside of home. This finding is in contrast with other studies. Li and co-workers reported that among adolescents in Taiwan males were more likely to be exposed to ETS than females [10]. Preston *et al*. in a study among Puerto Rican children found no significant difference between males and females in terms of exposure to ETS [11]. However the previously mentioned finding, if confirmed by future studies, has to be addressed by future research in order to clarify why this difference between sexes in exposure to ETS does exist. Multivariate analysis documented those adolescents whose parents and close friends were smokers were more likely to be exposed to passive smoking both at home and in public places. Nevertheless, it should be stressed that regarding ETS exposure at home the effect of parental smoking status was stronger than that of close friends smoking status. Thus the parents smoking habit is a strong determinant of ETS exposure at home. On the contrary considering ETS exposure outside home, an inverse picture emerged with close friends smoking to be a stronger predictor of passive smoking outside of home than parental smoking status.

The present study has several limitations. Firstly, it is a questionnaire study; there is a potential for information bias to occur. In addition, we did not use biomarkers of exposure to ETS in order to assess the exposure status of the participants. It has been reported that the assessment of exposure status to passive smoking by the use of both questionnaires and biomarkers led to results that differed significantly [11]. The questionnaires are very useful in order to provide information on the population potentially susceptible to exposure to secondhand smoke, while the biomarker reflects the amount of tobacco smoke inhaled, and thus is a more reliable index of exposure. A second limitation is that our study was school-based; therefore the sample is not entirely representative of all adolescents in Greece.

## 5. Conclusions

In conclusion our study documented a high prevalence of exposure to ETS (both at home and outside home) among a national sample of school-going adolescents in Greece. Gender (except for exposure to ETS outside home), age, and smoking status of parents were independent predictors of exposure to secondhand smoke. These findings could have some implications in regard to public health interventions aiming to control ETS exposure among adolescents. In particular, in order to reduce ETS exposure at home special attention should be paid to females, those with age ≥15 years, and those having parents, and close friends who smoke cigarettes. In regard to exposure to second hand smoke outside home public health initiatives should target adolescents with age ≤14 years, and those having parents, and close friends who smoke cigarettes. It should be underlined that a New Greek anti-smoking legislation came into effect from July 1, 2009. It is expected that this legislation will significantly reduce the adolescent’s burden of exposure to secondhand smoke outside home. However, exposure to ETS at home could not be effectively addressed only by legislation. Educational interventions targeting parents—especially those who are smokers could substantially reduce the exposure of adolescents to second hand smoke at home.

## Figures and Tables

**Table 1.** Selected Demographic Characteristics of Greek Nonsmoker Teenagers aged 11−17 years old (2004−2005).

Characteristic	**Males % (n)**	**Females % (n)**	**Total % (n)**
	50.7 (2589)	49.2 (2590)	100 (5179)
Age			
11–13 14 15 16–17	44.4 (1109) 29.6 (806) 21.3 (570) 4.7 (104)	41.1 (1044) 33.2 (878) 22.6 (592) 3.2 (76)	42.8 (2153) 31.3 (1684) 21.9 (1162) 4.0 (180)
Parents smoking			
None Both parents Father only Mother only	36.3 (903) 24.8 (679) 27.3 (680) 11.5 (305)	32.5 (819) 29.2 (756) 26.6 (685) 11.7 (320)	34.4 (1722) 27.0 (1435) 27.0 (1365) 11.6 (625)
Friends smoking			
None Some Most or all	61.3 (1579) 33.4 (855) 5.4 (138)	67.6 (1735) 27.2 (720) 5.2 (129)	64.4 (3314) 30.3 (1575) 5.3 (267)
In favor of banning smoking in public places			
Yes No	89.7 (2300) 10.3 (276)	88.2 (2279) 11.8 (300)	89.0 (4579) 11.0 (576)
ETS exposure			
At home Outside of the home Both home and outside of the home	76.8 (1917) 38.5 (965) 72.9 (666)	81.9 (2083) 37.9 (960) 77.1 (730)	79.3 (4000) 38.2 (1925) 75.1 (1396)

**Table 2.** Variables Associated with Exposure to Environmental Tobacco Smoke (ETS) among Greek Teenagers aged 11−17 years old. Univariate (OR [95% CI]) and Multivariate (AOR [95% CI]) analyses.

**Variable**	**Home**		**Outside of the home**	
	OR [95% CI]*	AOR [95% CI]**	OR [95% CI]*	AOR [95% CI]**
Age (years)				
11–13 14 15 16–17	1.00 1.05 [0.90, 1.23] **1.54 [1.27, 1.86]** **1.37 [1.00, 2.05]**	1.00 1.02 [0.87, 1.19] **1.43 [1.20, 1.72]** **1.29 [1.13, 2.18]**	1.00 0.85 [0.75, 1.07] **0.68 [0.58, 0.79]** **0.58 [0.42, 0.81]**	1.00 0.89 [0.79, 1.02] **0.75 [0.64, 0.87]** **0.68 [0.48, 0.96]**
Gender				
Female Male	1.00 **0.72 [0.64, 0.82]**	1.00 **0.72 [0.62, 0.81]**	1.00 1.02 [0.91, 1.14]	1.00 1.04 [0.93, 1.17]
Parents smokers				
None Both parents Father only Mother only	1.00 **2.97 [2.47, 3.57]** **2.06 [1.73, 2.45]** **2.46 [1.93, 3.13]**	1.00 **2.86 [2.35, 3.32]** **2.08 [1.76, 2.46]** **2.34 [1.87, 2.94]**	1.00 **1.45 [1.26, 1.68]** **1.24 [1.08, 1.44]** **1.27 [1.05, 1.53]**	1.00 **1.36 [1.18, 1.56]** **1.22 [1.06, 1.41]** **1.18 [1.00, 1.42]**
Close friends smokers				
No Some Most or all	1.00 **1.47 [1.25, 1.71]** **1.69 [1.19, 2.38]**	1.00 **1.21 [1.00, 1.41]** **1.47 [1.12, 1.53]**	1.00 **1.63 [1.44, 1.85]** **2.57 [1.92, 3.44]**	1.00 **1.49 [1.32, 1.69]** **2.90 [2.01, 4.07]**

* Unadjusted odds ratios (OR) with 95% Confidence Interval (CI);

** Adjusted odds ratio (AOR) with 95% Confidence Interval (CI).
